# Subanalyses in HFmrEF Patients of Asian Ethnicity

**DOI:** 10.1016/j.jacasi.2025.10.003

**Published:** 2025-12-02

**Authors:** Xin Du

**Affiliations:** Center of Heart Failure and Cardiomyopathy, Beijing Anzhen Hospital, the Capital Medical University, Beijing, China

**Keywords:** Asian Ethnicity, Heart Failure with Mildly Reduced Ejection Fraction (HFmrEF), Subanalyses

This presentation provides a comprehensive overview of heart failure with mildly reduced ejection fraction (HFmrEF), situating it within the broader spectrum of heart failure and highlighting its unique characteristics, clinical behavior, and management strategies (Video).

HFmrEF is defined by a left ventricular ejection fraction (LVEF) between 40% and 49%, occupying the middle ground between 2 well-established categories:^1^

• Heart failure with reduced ejection fraction (HFrEF), defined as LVEF ≤40%, where multiple therapies have demonstrated efficacy in major trials, and
• Heart failure with preserved ejection fraction (HFpEF), defined as LVEF ≥50%, a definition originally intended to provide a clear contrast to HFrEF.

HFmrEF accounts for approximately 14% to 25% of all heart failure cases.^1^ Importantly, the terminology “mildly reduced” is now favored over the earlier “midrange.” This shift is not merely semantic but conceptual, recognizing that an ejection fraction within this range may not always reflect established heart failure; it can also indicate early cardiomyopathy, residual myocardial damage from a small infarction, or subtle ventricular impairment often associated with chronic coronary syndrome.

The clinical profile of HFmrEF is hybrid in nature. Registry data show similarities with HFpEF in terms of older age, higher body mass index, and increased prevalence of hypertension and atrial fibrillation. In contrast, HFmrEF patients resemble those with HFrEF in sex distribution (more likely to be men) and higher prevalence of ischemic heart disease. These observations have been confirmed in Asian populations, such as the SCDB-HF (Singapore Cardiac Databank Heart Failure) Registry,^2^ a nationwide prospective study including all consecutive heart failure admissions across Singapore’s public hospitals.

Prognostically, HFmrEF is not benign. A U-shaped relationship between LVEF and all-cause mortality has been reported, with the lowest risk at an ejection fraction of 60% to 65%. Any deviation—whether below or above this range—is associated with increased mortality.^3^ Moreover, HFmrEF is a dynamic state rather than a fixed category. The CHART-2 (Chronic Heart Failure Analysis and Registry in the Tohoku District-2) Study demonstrated that at 1 year, 44% of patients with baseline HFmrEF transitioned to HFpEF, while 16% shifted to HFrEF, underscoring that patients can move in either direction over time.^4^

Management of HFmrEF is further complicated by variability in LVEF measurement, particularly with echocardiography, which can lead to misclassification between HFpEF, HFmrEF, and HFrEF, and introduces challenges in applying trial evidence.

Despite these challenges, a growing body of data informs pharmacological management. The overarching principle advocated is to treat HFmrEF in a manner analogous to HFrEF. Individual patient-level meta-analyses of randomized, double-blind trials demonstrate consistent benefits across the LVEF spectrum for several key drug classes. Notably, SGLT2 inhibitors, Finerenone (a nonsteroidal mineralocorticoid receptor antagonist), sacubitril/valsartan (ARNI), and beta-blockers all show efficacy extending into the HFmrEF range.^5^, ^6^, ^7^, ^8^

Based on this evidence, the following treatment recommendations are proposed:

• SGLT2 inhibitors are strongly recommended as foundational therapy.
• Mineralocorticoid receptor antagonists (MRAs) are also recommended.
• For patients requiring renin–angiotensin system blockade, ARNI (sacubitril/valsartan) should be preferred over angiotensin-converting enzyme inhibitors or angiotensin receptor blockers.
• Beta-blockers are recommended, particularly in patients with sinus rhythm.

In summary, HFmrEF is a distinct, prevalent, and clinically important intermediate phenotype of heart failure. It combines features of both HFrEF and HFpEF, demonstrates substantial transition over time, and carries significant prognostic risk. Current evidence supports a proactive therapeutic approach—mirroring that of HFrEF—with SGLT2 inhibitors, MRAs, ARNI, and beta-blockers forming the cornerstone of management to improve outcomes in this sizable patient population.

## Funding Support and Author Disclosures

The author has reported that he has no relationships relevant to the contents of this paper to disclose.

## Appendix

Video 1 Subanalyses in HFmrEF Patients of Asian Ethnicity
